# Optical absorption microscopy of localized atoms at microwave domain: two-dimensional localization based on the projection of three-dimensional localization

**DOI:** 10.1038/s41598-019-57141-z

**Published:** 2020-01-17

**Authors:** Bibhas Kumar Dutta, Pradipta Panchadhyayee, Indranil Bayal, Nityananda Das, Prasanta Kumar Mahapatra

**Affiliations:** 1 Department of Physics, Sree Chaitanya College (WB State University), Habra, North 24 Parganas, 743 268 W. B., India; 2 Department of Physics (UG & PG), Prabhat Kumar College, Contai (Vidyasagar University), Purba Medinipur, 721 401 W. B., India; 3 Gopinathpur High School (HS) Gopinathpur, Purba Medinipur, 721 633 W. B., India; 4 Department of Physics, J. K. College (Sidho Kanho Birsha University), Purulia, 723 101 W. B., India; 5 ITER, Siksha ‘O’ Anusandhan University, Bhubaneswar, 751030 Odisha India

**Keywords:** Quantum optics, Scanning probe microscopy

## Abstract

*A new approach for achieving two* – *dimensional* (*2D*) *atom localization microscopy based on the projection of three* – *dimensional* (*3D*) *localization in the plane of the detector* is described in the present work. Spatial variation of the position-dependent 2D-localization pattern in the *xy*-plane is obtained with the shifting of the position of the detector along the z-axis under the parallel- and cross- axis configurations of the standing-wave fields. An attempt is made to study the 2D-localization characteristics in the specific parametric conditions for which the localization structures evolve with different shapes eventually leading to 100% detection probability of the atom both in the sub-wavelength and sub-half-wavelength regimes. The scope of tuning the cross-axis configuration over a wide range adds novelty and robustness to this model. Apart from the 2D-localization, various localization patterns with eight- to single-peak structures appear as interesting outcomes through the efficient manipulation of control parameters in the study of one-dimensional (1D) atom localization. The application of the traveling-wave field or its equivalent appears to be unique in achieving high-precision localization with maximal probability (100%) in both the 1D and 2D field-configuration schemes. Proper tuning of the traveling wave accompanied by the standing wave in the 1D scheme results in the single-peak localization in the sub-half-wavelength range. As a whole, the present work seems to be very much efficient for high-precision optical lithography.

## Introduction

Precision atomic position measurement^1–5^ has gained momentum with the use of spatially modulated coherence effect induced by the laser field. Different fields of studies like atom nano-lithography^6^, laser cooling and trapping of neutral atoms^7^ and Bose-Einstein condensation^8^ have been enriched by the versatile applications of atom localization. The pioneering works^2,3,9,10^ on atom localization involving energy and phase shifts with spatial variation are followed by studies in one dimension^11–18^, which describe the fundamental aspects of measurement-induced localization of atoms through a variety of atom-field configurations. To be more specific, an interesting property of coherent population trapping (CPT) is exploited in the study of atom localization^14^. The mechanism of electromagnetically induced transparency (EIT) is also taken into consideration in manipulating the localization pattern^11,18^. As explored in refs. ^12,13^, the phenomenon of phase coherence under closed-loop interaction scheme appears to be the main controlling unit in the process of atom localization. High-precision localization exhibited in refs. ^15,17^ is attributed to the effect of dynamic phase modulation in the pump-probe resonance. The 1D-localization based works mainly highlight effective control and desired manipulation of the localization peaks in sub-wavelength, or sub-half-wavelength domain. The techniques of atom localization based on single standing-wave formation in the 1D cases have been extended to the study of 2D (3D) localization with the configuration of standing waves along the two (three) orthogonal directions^19–33^. Much attention has been paid in such earlier works on 2D localization with the spatial modulation of absorption and transparency of the weak probe field. Due to the impact of quantum coherence emerging out of the atom-field interactions in different types of atomic systems under the standing-wave regime, different localization structures with interesting spatial patterns in a plane have been reported.

**Figure 1:**
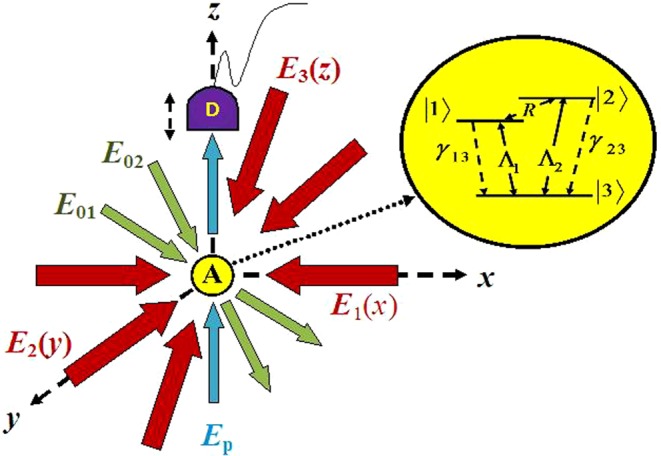
Schematic presentation of field configuration: ${E}_{S}(S=1,2,3)$ are the three standing-wave fields. ${E}_{p}$ and ${E}_{0S}$ stand for the probe and traveling-wave fields, respectively. Here $A$ (inset: corresponding atomic system) and $D$ are the atom and the detector, respectively.

In this article, we have studied the atom localization based on dynamic Stark effect in the absorptive response in a *V*-type atom driven by the spatially modulated microwave field. Similar low-frequency-induced control of coherence effects are reported to be of increasing demand in various schemes at molecular level^34^, superconducting quantum circuits^35^ and off-resonantly coupled quantum dot-cavity systems^36^. In the present atom-field system, we have shown the coherent control of localization in both the 1D and 2D cases for different choices of parameters controlling the evolution of standing waves in the system. In the following, we present the salient features of our study. (I) Various 1D-localization patterns with eight- to single peak structures are achieved by suitable tuning of control parameters when a traveling wave is subjected to the system. It has been shown that the role of traveling wave is to produce a significant shift of the localization peaks. (II) The single-peak localization is found to be attainable with 100% detection probability of the atom within sub-half-wavelength range for a typical choice of values of Rabi frequencies of the traveling and standing waves. Single-peak localization in the sub-half-wavelength range is achieved without incorporating any phase shift. (III) Apart from Rabi frequencies, modifications in the appearance of localization peaks are shown to be possible by controlling the detuning parameters involved in the system. The Rabi-induced shifting and manipulation of localization peaks are prominently noticeable in the present work in comparison to the usual detuning-induced control of localization. (IV) Akin to the aims concerned with the 1D scheme, a study on 2D localization is presented. The cross-axis configuration with a third standing-wave field in the *z*-direction along with two standing-wave fields in the resonant condition shows uniqueness in achieving 100% probability of detection of the atom in the sub-wavelength regime when scanned by the detector along the *z*- axis, which has been shown as an equivalent technique in detecting atom under 3D-projection-based 2D-localization schemes in the *xy* plane. It requires proper positioning of the detector along the *z*-axis (Fig. 1), which is unique when compared to that reported in ref. ^25^. Tolerance of parameter values, related to the cross-axis configuration, adds to the novelty of the proposed model. (V) For the parallel-axis configuration with two orthogonal non-resonant standing wave fields, we explore the specific parametric conditions for which the 2D localization characteristics shape in different localization structures and ensure 100% detection probability in the sub-half-wavelength region. Both ways of detection can be treated as fascinating knobs within the scope of the present work. Overall, the model presented in this work is suitable for potential application in high-precision optical lithography^24^.

## Theoretical Formulation

We consider an ideal three-level atom interacting with a microwave field of frequency $\omega$ as shown in Fig. 1. The spontaneous decay from the excited levels $\mathrm{|1}\rangle$ and $\mathrm{|2}\rangle$ to the ground state $\mathrm{|3}\rangle$ are designated by the decay rate ${\gamma }_{13}$ and ${\gamma }_{23}$, respectively. If ${\epsilon }_{i}$ ($i=1,2,3$) be the corresponding energies of the energy levels, we have the atomic transition frequencies ${\omega }_{13}=({\epsilon }_{1}-{\epsilon }_{3})$/$\hslash$, ${\omega }_{21}=({\epsilon }_{2}-{\epsilon }_{1})$/$\hslash$ and ${\omega }_{23}=({\epsilon }_{2}-{\epsilon }_{3})$/$\hslash$. Under the electric dipole approximation, the strength of coupling of the atom with the coherent field is denoted by the Rabi frequency

$$R=\frac{{\bar{\mu }}_{12}.[{\bar{E}}_{0x}\,{\sin }(kx+{\varphi }_{1})+{\bar{E}}_{0y}\,{\sin }(ky+{\varphi }_{2})+{\bar{E}}_{0z}\,{\sin }(kz+{\varphi }_{3})]}{2\hslash }$$

for the coherent field

$$\bar{E}(t)=\frac{[{\bar{E}}_{\mathrm{\varphi }x}\,{\sin }(kx+{\varphi }_{1})+{\bar{E}}_{0y}\,{\sin }(ky+{\varphi }_{2})+{\bar{E}}_{0z}\,{\sin }(kz+{\varphi }_{3})]}{2}{e}^{i\omega t}+c.\,c\mathrm{.}.$$

Here, the atomic transition moment ${\bar{\mu }}_{12}$ is involved in the transition $\mathrm{|1}\rangle \leftrightarrow \mathrm{|2}\rangle$ and ${\varphi }_{j}$ $(\,j=1,2,3)$ implies the phase imparted from the configuration of standing-wave field formation. Two incoherent pump fields are considered to be operating in the transitions $\mathrm{|1}\rangle \leftrightarrow \mathrm{|3}\rangle$ and $\mathrm{|2}\rangle \leftrightarrow \mathrm{|3}\rangle$ with the pumping rates ${\Lambda }_{1}$ and ${\Lambda }_{2}$ respectively. In the interaction picture, the Hamiltonian giving rise to the time-evolution dynamics of the system is given by using the rotating-wave-approximation (RWA) as

1 $$H=-\,\hslash [\,-\,\Delta |2\rangle \langle 2|+(R(x,y,z)|1\rangle \langle 2|+c.\,c.\,)]$$

where the detuning term Δ = $\omega -{\omega }_{21}$.

The dynamical behaviour of the system under consideration can be represented by the time evolution of the density-matrix operator

2 $$\frac{\partial \rho }{\partial t}={(\frac{\partial \rho }{\partial t})}_{reversible}+{(\frac{\partial \rho }{\partial t})}_{irreversible}$$

where the reversible part represents the interaction between the coherent field and the atom, which is given by

3 $${(\frac{\partial \rho }{\partial t})}_{reversible}=-\,\frac{i}{\hslash }[H,\rho ].$$

In order to introduce the effect of dissipative process in the system, we represent

4 $$\begin{array}{rcl}{(\frac{\partial \rho }{\partial t})}_{irreversible} & = & {(\frac{\partial \rho }{\partial t})}_{spon.damping}+{(\frac{\partial \rho }{\partial t})}_{inco.pump}\\  & = & -\,\sum _{j=1,2}\,\frac{{\gamma }_{j3}}{2}[\{|3\rangle \langle 3|,\rho \}-2|3\rangle \langle j|\rho |j\rangle \langle 3|]\\  &  & -\,\frac{{\Lambda }_{1}}{2}\,\sum _{j=1,3}\,[\{|j\rangle \langle j|,\rho -\sum _{k(\ne j)=1,3}\,2|j\rangle \langle k|\rho |k\rangle \langle j|]\\  &  & -\,\frac{{\Lambda }_{2}}{2}\sum _{j=2,3}\,[\{|j\rangle \langle j|,\rho -\sum _{k(\ne j)=2,3}\,2|j\rangle \langle k|\rho |k\rangle \langle j|].\end{array}$$

The set of equations dealing with the time derivative of the density matrix elements appear as:

5 $${\dot{\rho }}_{11}=iR(x,y,z){\rho }_{21}-iR{(x,y,z)}^{\ast }{\rho }_{12}-({\Lambda }_{1}+{\gamma }_{13}){\rho }_{11}+{\Lambda }_{1}{\rho }_{33}$$

6 $${\dot{\rho }}_{22}=iR{(x,y,z)}^{\ast }{\rho }_{12}-iR(x,y,z){\rho }_{21}-({\Lambda }_{2}+{\gamma }_{23}){\rho }_{22}+{\Lambda }_{2}{\rho }_{33}$$

7 $${\dot{\rho }}_{33}=({\Lambda }_{1}+{\gamma }_{13}){\rho }_{11}+({\Lambda }_{2}+{\gamma }_{23}){\rho }_{22}-({\Lambda }_{1}+{\Lambda }_{2}){\rho }_{33}$$

8 $${\dot{\rho }}_{21}=-\,({\Gamma }_{21}+i\Delta ){\rho }_{21}-iR{(x,y,z)}^{\ast }({\rho }_{22}-{\rho }_{11})$$

9 $${\dot{\rho }}_{31}=-\,{\Gamma }_{31}{\rho }_{31}-iR{(x,y,z)}^{\ast }{\rho }_{32}$$

10 $${\dot{\rho }}_{32}=-\,({\Gamma }_{32}-i\Delta ){\rho }_{32}-iR(x,y,z){\rho }_{31}$$

where ${\Gamma }_{21}=({\Lambda }_{1}+{\Lambda }_{2}+{\gamma }_{13}+{\gamma }_{23})/2$, ${\Gamma }_{31}={\Lambda }_{1}+({\Lambda }_{2}+{\gamma }_{13}\mathrm{)/2}$ and ${\Gamma }_{32}={\Lambda }_{2}+({\Lambda }_{1}+{\gamma }_{23}\mathrm{)/2}$. In the present model, we have chosen ${\gamma }_{13}={\gamma }_{23}=\gamma$.

In the present atomic system, we have considered the presence of a weak laser field that probes the coherence effect in its absorptive response for the atom being driven by a microwave field. We note that the absorption of the probe field is tunable over the transitions from $\mathrm{|3}\rangle$–$\mathrm{|1}\rangle$ to $\mathrm{|3}\rangle$–$\mathrm{|2}\rangle$. To find the susceptibility under linear response theory based on quantum regression theorem, we have followed the traditional approach as described in refs. ^17,37^. The linear susceptibility $\chi ({\omega }_{p})$ of the probe field of frequency ${\omega }_{p}$ is given in terms of the Fourier transform of the average value of the two-time commutator of the atomic operator as^17,37^

11 $$\chi ({\omega }_{p})=i\frac{2n}{{\varepsilon }_{0}\hslash }\,\sum _{j=1,2}\,|{\mu }_{j3}{|}^{2}\,{\int }_{0}^{\infty }\,{\langle [{\sigma }_{j3}(\tau ),{\sigma }_{j3}^{\dagger }(0)]\rangle }_{s}{e}^{-i({\omega }_{j3}-{\omega }_{p})\tau }d\tau $$

where we represent the atomic-transition operator as the lowering operator ${\sigma }_{ij}=|i\rangle \langle j|$ and the raising operator ${\sigma }_{ij}^{\dagger }=|j\rangle \langle i|$ for $|j\rangle$ being the higher state in comparison to the state $|i\rangle$. In the correlation function, the transition operator is considered in the interaction picture representation. ${\mu }_{j3}$ denotes the transition dipole moment and $n$ is the atomic density. The index $s$ indicates the steady state of the system. In order to determine the two-time commutator functions, we first redefine the density-matrix elements in the set of Eqs. (5–10) in terms of atomic-transition operators, then solve the new set of equations in Laplace space to find the two-time correlation function by using quantum regression theorem^37^. Finally, we obtain the average values of two-time commutators as required in expression (11) to find the position dependent complex susceptibility $\chi ({\omega }_{p},x,y,z)$ whose imaginary part provides the coefficient of spatially modulated absorption in the standing-wave regime and is given by

12 $${\mathscr{A}}={{\mathscr{A}}}_{0}F(x,y,z)$$

where ${{\mathscr{A}}}_{0}$ stands for the constant factor of expression (11) and $F(x,y,z)$ is the Filter function representing the position distribution of atomic absorption expressed as:

13 $$\begin{array}{rcl}F(x,y,z) & = & {Im}{[i\tfrac{(\tau +{\Gamma }_{32}+i\Delta )({\rho }_{33}(0)-{\rho }_{11}(0))-iR(x,y,z){\rho }_{21}(0)}{(\tau +{\Gamma }_{31})(\tau +{\Gamma }_{32}+i\Delta )+|R(x,y,z){|}^{2}}]}_{(\tau =i{\delta }_{-})}\\  &  & +\,{Im}{[i\tfrac{(\tau +{\Gamma }_{31})({\rho }_{33}(0)-{\rho }_{22}(0))-iR{(x,y,z)}^{\ast }{\rho }_{12}(0)}{(\tau +{\Gamma }_{31})(\tau +{\Gamma }_{32}+i\Delta )+|R(x,y,z){|}^{2}}]}_{(\tau =i{\delta }_{+})}\end{array}$$

with ${\rho }_{21}(0)=\frac{iR{(x,y,z)}^{\ast }({\rho }_{11}(0)-{\rho }_{22}(0))({\Gamma }_{21}-i\Delta )}{{\Gamma }_{21}^{2}+{\Delta }^{2}}$ and ${\rho }_{12}\mathrm{(0)}={\rho }_{21}^{\ast }\mathrm{(0)}$. The detuning parameters ${\delta }_{\pm }=\delta \pm {\omega }_{0}$ where ${\omega }_{0}={\omega }_{21}\mathrm{/2}$ and $\delta ={\omega }_{m}-{\omega }_{p}$ with ${\omega }_{m}={\omega }_{13}+{\omega }_{0}={\omega }_{23}-{\omega }_{0}$. The Rabi frequencies $R(x,y,z)$ of the fields are taken as position dependent. For 2D (1D) localization, spatial variation of the field components along the *z*− (*y*−, *z*−) direction(s) is usually not taken into account.

The steady-state values of populations are determined by the expressions given as follows

14 $${\rho }_{11}(0)=\frac{{\Lambda }_{1}({\Lambda }_{2}+{\gamma }_{23})+({\Lambda }_{1}+{\Lambda }_{2})f}{(\,f+2{\Lambda }_{1}+{\gamma }_{13})(\,f+2{\Lambda }_{2}+{\gamma }_{32})-(\,f-{\Lambda }_{1})(\,f-{\Lambda }_{2})},$$

15 $${\rho }_{22}(0)=\frac{{\Lambda }_{2}({\Lambda }_{1}+{\gamma }_{13})+({\Lambda }_{1}+{\Lambda }_{2})f}{(\,f+2{\Lambda }_{1}+{\gamma }_{13})(\,f+2{\Lambda }_{2}+{\gamma }_{32})-(\,f-{\Lambda }_{1})(\,f-{\Lambda }_{2})},$$

and

16 $${\rho }_{33}(0)=1-{\rho }_{11}(0)-{\rho }_{22}(0)$$

with $$f=2{\Gamma }_{21}|R(x,y,z{)|}^{2}/({\Gamma }_{21}^{2}+{\Delta }^{2})$$.

We must mention that, for the atom moving through the standing-wave fields, atom-field interaction energy is taken to be highly dominant over the kinetic energy of its centre-of-mass motion under the Raman-Nath approximation^18^ i.e. the recoil of the atom during its passage through the standing-wave regime is neglected. Due to this reason the kinetic-energy term is not included in the Hamiltonian (expression (1)) and the probability corresponding to the centre-of-mass wave function ${\psi }_{CM}$ of the atom can be considered as stationary. Viability of such approximation is confirmed in the formation of atomic gratings at Raman-Nath diffraction regime^38,39^.

By following the approach, as derived in the earlier work^40^, the conditional position probability ($W(x,y,z)$) of finding the atom, when the signal carried by the probe beam is absorbed by the detector atom, gives rise to the following ansatz:

$$W(x,y,z)\approx |N^{\prime} {|}^{2}|{\psi }_{CM}{|}^{2}{f}_{D}(x,y,z),$$

where *N*′ is the normalization constant and ${f}_{D}(x,y,z)$ is the Detector Response Function^40^ which can be expressed as:

$${f}_{D}(x,y,z)\approx {C}_{0}{\mathscr{A}}(x,y,z),$$

*C*_0_ being the proportionality constant. Taking into account the Eq. (12) the conditional position probability appears as

17 $$W(x,y,z)=|N{|}^{2}|{\psi }_{CM}{|}^{2}F(x,y,z),$$

where $N=N^{\prime} \sqrt{{C}_{0}\,{A}_{0}}$. Thus, the spatial dependence of conditional position probability manifests through the Filter function $F(x,y,z)$, which, in turn, corresponds to the probe absorption spectrum, and thereby leads to position information of the atom under microscopic measurement of probe absorption.

For the study of 1D localization of the atom in the standing-wave regime formed along the *x*- direction, we consider the presence of a traveling wave with a standing wave, which needs to redefine the position-dependent Rabi frequency as

18 $$R(x)={R}_{0}+{R}_{1}\,{\sin }({k}_{1}x+{\varphi }_{1})$$

Here, the Rabi frequency ${R}_{0}$ stands for the coherent mixing of two traveling waves as shown in Fig. 1. The standing-wave field ${R}_{1}(x)$ can be directly produced by the counter-propagating wave-vector of the field with Rabi frequencies ${R}_{1}$ along the *x*- direction and ${k}_{1}$ being the propagation vector. Phase-shift introduced by the formation of standing wave component is denoted by ${\varphi }_{1}$.

To investigate 3D-projection-based 2D localization of the atom, we consider two distinct spatial field configurations with two standing-wave fields: (i) parallel-axis and (ii) cross-axis.

For the *parallel*-*axis configuration* with two *orthogonal* standing-wave fields the position dependent Rabi frequency for the study of 2D atom localization can be written as

19 $$R(x,y)={R}_{0}+{R}_{1}\,{\sin }({k}_{1}x+{\varphi }_{1})+{R}_{2}\,{\sin }({k}_{2}\,y+{\varphi }_{2}).$$

According to the spatial field arrangement with the *cross*-*axis configuration*, the actual standing-wave fields need not to be purely orthogonal. Only, the components of those fields along the *x*- and *y*- directions are to be considered. Thus, we have the choice to define the Rabi frequencies in the following form:

$$R(x,y)={R}_{0}+{R}_{1}\,\sin ({k}_{1x}x+{\varphi }_{1})+{R}_{2}\,\sin ({k}_{2y}y+{\varphi }_{2})$$

Here, ${k}_{1x}={k}_{1}\,cos\,{\theta }_{1}$ (${k}_{2y}={k}_{2}\,cos\,{\theta }_{2}$) for ${\theta }_{1}$ (${\theta }_{2}$) being the respective angular separation of the wave-vector from the *x*- (*y*-) direction. For simplicity of notation, we replace the factor $cos\,{\theta }_{j}(j=1,2)$ in the respective terms with ${k}_{1x}$ and ${k}_{2y}$ by ${\eta }_{j}$ to redefine non-zero and finite ${k}_{1x}={\eta }_{1}{k}_{1}$ and ${k}_{2y}={\eta }_{2}{k}_{2}$ for $0 < {\eta }_{j}\le 1$. So the above equation can be simply expressed as:

20 $$R(x,y)={R}_{0}+{R}_{1}\,\sin ({\eta }_{1}{k}_{1}x+{\varphi }_{1})+{R}_{2}\,\sin ({\eta }_{2}{k}_{2}y+{\varphi }_{2})$$

We must note that constant traveling-wave field $({R}_{0})$ can be replaced by a standing wave field in a cross-axis (see the standing-wave field formation along $z$-axis in Fig. 1)-configuration. In accordance with the standing-wave representation in the expression (20) it is here represented by ${R}_{3}\,sin({\eta }_{3}{k}_{3}z+{\varphi }_{3})$. Without loss of generality, keeping the objectives of the present study in view, the spatial phase, ${\varphi }_{3}$, is set to zero. It is worth noting that, for a particular value of the Rabi frequency ${R}_{3}$, we have to choose a typical value of ${\eta }_{3}{k}_{3}z$, so that the detector placed at a certain position along the *z*-axis can detect the spatially modulated probe beam in the *xy*-plane, when the beam is coming towards it after passing through the atomic ensemble under the interaction with the standing waves (see the detector (D) arrangement in Fig. 1). It is obvious that the use of ${R}_{0}$ is a pre-requisite, but quite optional for its formation in terms of coherent mixing of traveling-wave fields in the present study.

### Momentum distribution function

**Figure 2:**
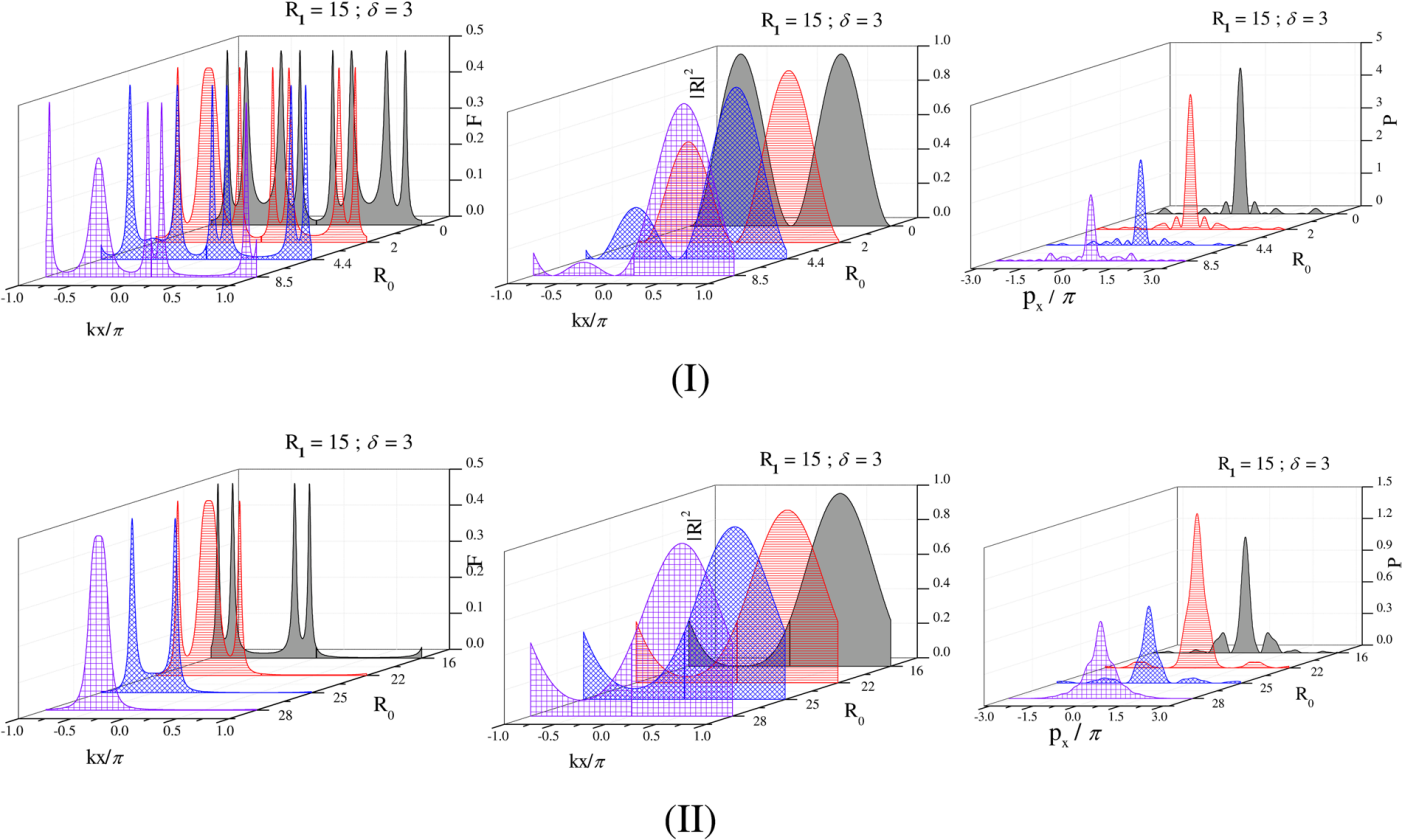
1D Filter function $F(x)$ (left panel), and the corresponding $R(x)$ (middle panel) as a function of $(kx/\pi )$, and 1D momentum distribution function P(p_x_) (right panel)as a function of p_x_/π in units of $\hslash /\lambda$:-(I) (from back to front of each graph): ${R}_{0}=0$ (black), ${R}_{0}=2$ (Red), ${R}_{0}=4.4$ (blue), ${R}_{0}=8.5$ (violet); (II) (from back to front of each graph): ${R}_{0}=16$ (black), ${R}_{0}=22$ (Red), ${R}_{0}=25$ (blue), ${R}_{0}=28$ (violet). Other parameters ${R}_{1}=15$, $\delta =3$, $\Delta =0$, ${\varphi }_{1}=0$, ${\gamma }_{23}=1$, ${\Lambda }_{1}=0.15$, ${\Lambda }_{2}=0.15$, ${\omega }_{0}=10$.

With the study of variation in position distribution of atoms, another important aspect is unveiled by observing correspoding change in the momentum distribution, which can be viewed as a natural consequence of Heisenberg uncertainty principle. Thus, one can ascertain the position information of an atom through the measurement on momentum distribution after interaction of the atom with the space-modulated field. As is well known, the occurrence of a peak in conditional position distribution of atoms results in the spreading of momentum distribution, which has been studied in a limited number of works^14,25^ in the recent past. In this regard, we depict the momentum distribution of atoms in Fig. 2 for each case of 1D and later for the two cases of 2D localization schemes, which are based on the corresponding position distributions. The following equations are respectively considered to compute the 1D and 2D momentum distribution functions

21 $$P({p}_{x})={|{\int }_{-\pi }^{\pi }\sqrt{W(x,{p}_{x})}{e}^{i{p}_{x}x}dx|}^{2}$$

and

22 $$P({p}_{x},{p}_{y})={|{\int }_{-\pi }^{\pi }{\int }_{-\pi }^{\pi }\sqrt{W(x,y,{p}_{x},{p}_{y})}{e}^{i{p}_{x}x}{e}^{i{p}_{y}y}dxdy|}^{2}$$

**Figure 3:**
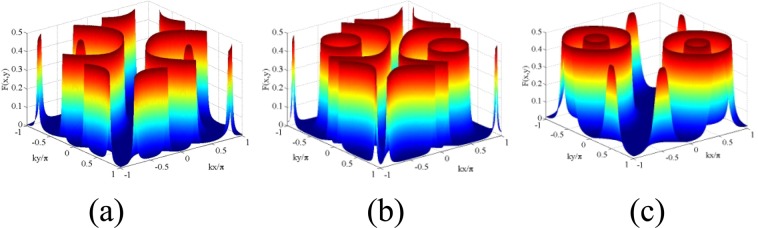
2D Filter function $F(x,y)$ as a function of $(kx/\pi ,ky/\pi )$: (**a**) ${R}_{1}={R}_{2}=15,\delta =20$; (**b**) ${R}_{1}={R}_{2}=15,\delta =15$; (**c**) ${R}_{1}={R}_{2}=7,\delta =3$. Other parameters ${\gamma }_{23}=1$, ${\Lambda }_{1}={\Lambda }_{2}=0.15$, ${\omega }_{0}=10$, ${\varphi }_{1}={\varphi }_{2}=0$, $\Delta =0$.

The integration is taken over a range of single wavelength of the standing wave in the *x*- and *y*- directions.

## Results and Discussions

In this section, we have presented spatially modulated absorption through the calculation of Filter function *F* (as given in Eq. (13)) for probe laser in the case of a three-level *V*-type atom using different standing-wave field arrangements. Measurement of the probe field absorption spectra also provides the position information of the atom interacting with the standing-wave fields for various values of the control parameters involved in the system. The peak position of the probe absorption occurs where the conditional position probability of atom becomes appreciable. Peak height of the probe absorption in one period of the standing-wave fields stands for the measure of localization probability, whereas peak width signifies the degree of precision of localization. The presentation of momentum distribution probability for the two extreme cases based on position information of atom is explained under the framework of position-momentum uncertainty. In our calculations, all the other parameters are scaled by the decay rate ${\gamma }_{13}$ taken as unity. The parameters ${\gamma }_{23}=1,{\Lambda }_{1}=0.15,{\Lambda }_{2}=0.15,{\omega }_{0}=10$ are kept invariant throughout the study. All ${k}_{j}(\,j=1,2)$ values are taken to be the same i.e., $k$, for all results given below.

### 1D atom localization

To explore the influence of control parameters of the 1D localization characteristics the 1D Filter function $F(x)$ under the resonant condition ($\Delta =0$) of field arrangement (Eq. (18)) is plotted in Fig. 2 (left panel) with the corresponding variation in momentum distribution function $P({p}_{x})$ in the right panel as a function of ${p}_{x}/\pi$ in different parameter conditions. We set ${R}_{1}=15$, $\delta =3$, and ${\varphi }_{1}=0$ for the purpose. In Fig. 2 we gradually increase the Rabi frequency of the traveling-wave field (${R}_{0}$) through a gradual increase in the intensity of laser associated with this field and present different patterns of 1D localization with a decreasing number of peaks from eight to single peak. To apprehend the changes in localization characteristics (left panels of Fig. 2) in a more convincing way we have shown the spatially modulated Rabi frequency regime i.e., the variation of resultant ${R}^{2}(x)$ in the middle panels of Fig. 2.

Our effort is to tune the control parameters for achieving the maximum number of localization peaks in the localization profile and then to reduce the number of peaks one by one in the profile to finally have the single-peak localization profile. In the present scheme, the coherent and incoherent fields collectively induce dynamic Stark splitting in the energy levels of the atomic system. Thus for all possible transitions, eight maximum peaks are expected to occur. Here, it is to note that, in the absence of laser responsible for the traveling-wave field (i.e., for ${R}_{0}$), eight-peak localization structure emerges in the one-wavelength standing wave regime with four localization peaks in each half period (Fig. 2(I)). The accuracy of space localization becomes maximum with a value of 1/8 in consonance with the precision so far reported^16^. For this case, the squared variation of resultant Rabi frequency (${R}^{2}(x)$) gives two maxima at the antinodes and minima at the central node along with the side nodes. Application of the laser associated with non-zero values of ${R}_{0}$ imprints its prominent signature in the variation of ${R}^{2}(x)$. With the gradual increase in ${R}_{0}$ the plot of ${R}^{2}(x)$ shows a gradual decrease in the height of maximum at the antinode of the left half period accompanied by the shifting of maxima at both halves. Due to a small decrement in height but increase in width of the left peak in the ${R}^{2}(x)$- variation for ${R}_{0}=2$ the eight-peak localization pattern evolves into a seven-peak localization structure with three peaks in the left half-wavelength region (−1 ≤ ${k}_{1}x$/*π* ≤ 0) and four peaks, as before, in the right half-wavelength region (0 ≤ ${k}_{1}x$/*π* ≤ 1). With a further increase of ${R}_{0}$ to 4.4, the peak-shifting effect gets prominence in the left half resulting in the appearance of only two localization peaks with greater separation in that half with a total number of six peaks in the whole wavelength regime. For the above cases (${R}_{0}=2$ and 4.4) four peaks segregate in two closely spaced pairs of localization peaks having the almost same width and height in the right half. But with the rise of ${R}_{0}$ the separation between paired structures gets increased. Out of the three localization peaks originated in the left half, −1 ≤ ${k}_{1}x$/*π* ≤ 0, the height of the middle peak is reduced appreciably for ${R}_{0}=8.5$ with the onset of another period near the left node in ${R}^{2}(x)$- variation. Two largely separated peaks occur in the right half (0 ≥ ${k}_{1}x$/*π* ≤ 1) due to the impact of a greater value of ${R}_{0}$ as is shown for the change in ${R}^{2}(x)$ profile in this half period. Momentum distribution probability $(P({p}_{x}))$ for all the above cases are plotted in the right panel of Fig. 2(I).

To further reduce the number of peaks we raise the intensity of traveling-wave field (${R}_{0}$) and plot the Filter function $F(x)$, ${R}^{2}(x)$ and $P({p}_{x})$ respectively in the left, middle and right panels of Fig. 2(II). In such absorption microscopy successive attainment of sub-half-wavelength localization with four, three, two and single peak is a very remarkable and interesting outcome only by proper tuning of ${R}_{0}$. We note that the emergence of such sub-half-wavelength localization can be easily apprehended by close inspection of the corresponding change in ${R}^{2}(x)$ profile. Importantly at the end, control of only the traveling-wave field with fixed Rabi frequency of the standing wave field and $\delta$ leads to 100% detection probability of the atom with the generation of a single localization peak in the sub-half-wavelength region of the standing-wave regime.

As is mentioned earlier, the measurement on momentum distribution can assure the state of position localization of the atom after its interaction with the spatially modulated field. To comprehend the localization more clearly, we present the 1D momentum distribution $P({p}_{x})$ for the parameters of Fig. 2 (left panel) in Fig. 2 (right panel). All the momentum-distribution graphs in Fig. 2 exhibit a central peak with maximal amplitude accompanied by side peaks of gradually diminishing amplitudes. The momentum-distribution graphs of Fig. 2 shows that the number of peaks, as well as peak width and relative height of the side peaks, decreases in the momentum profile of the atom whose position localization is of less precision. The narrowing in the Filter function of the localization of atoms is expected to result in the widening of momentum distribution. So these graphs are in agreement with the uncertainty relation. Further, the nature of momentum distribution implies that the recoil kinetic energy of the atom is limited by the field-dependent interaction energy of the atom in the standing wave regime ensuring the validity of Raman-Nath approximation in our chosen parameters’ regime.

### 2D atom localization

**Figure 4:**
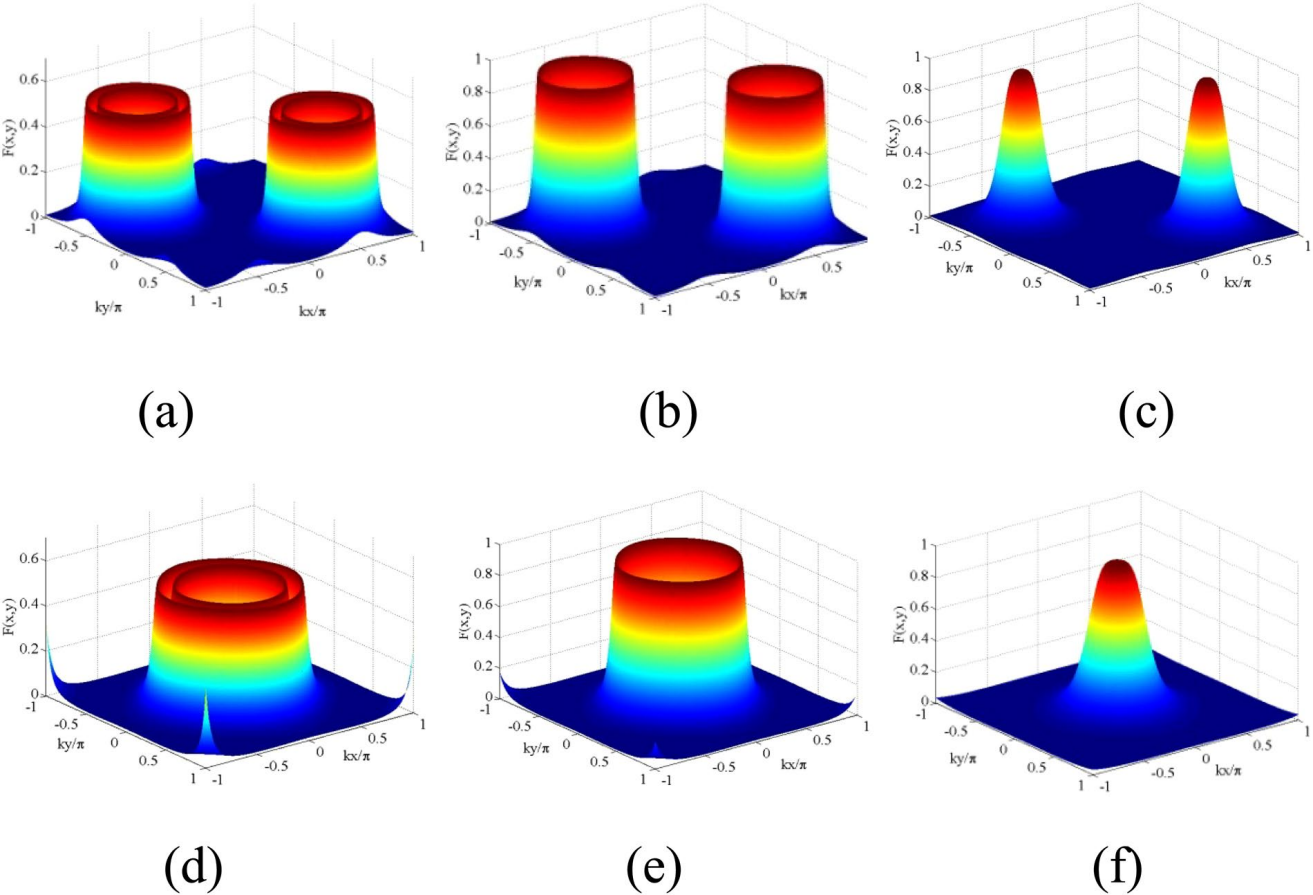
2D Filter function $F(x,y)$ as a function of $(kx/\pi ,ky/\pi )$: (**a**) ${R}_{1}={R}_{2}=7,\delta =1$; (**b**) ${R}_{1}={R}_{2}=7,\delta =0$; (**c**) ${R}_{1}={R}_{2}=5,\delta =0$. Other parameters for (**a**–**c**) ${\varphi }_{1}={\varphi }_{2}=0$. (**d**–**f**) Have the conditions ${\eta }_{1}={\eta }_{2}=0.7;{\varphi }_{1}={\varphi }_{2}=\pi /2$ or −$\pi \mathrm{/2}$ and correspond to other parameter conditions same as those of (**a**–**c**), respectively. Other parameters for all the graphs: ${\gamma }_{23}=1,{\Lambda }_{1}=0.15={\Lambda }_{2}=0.15,{\omega }_{0}=10,\Delta =0$.

**Figure 5:**
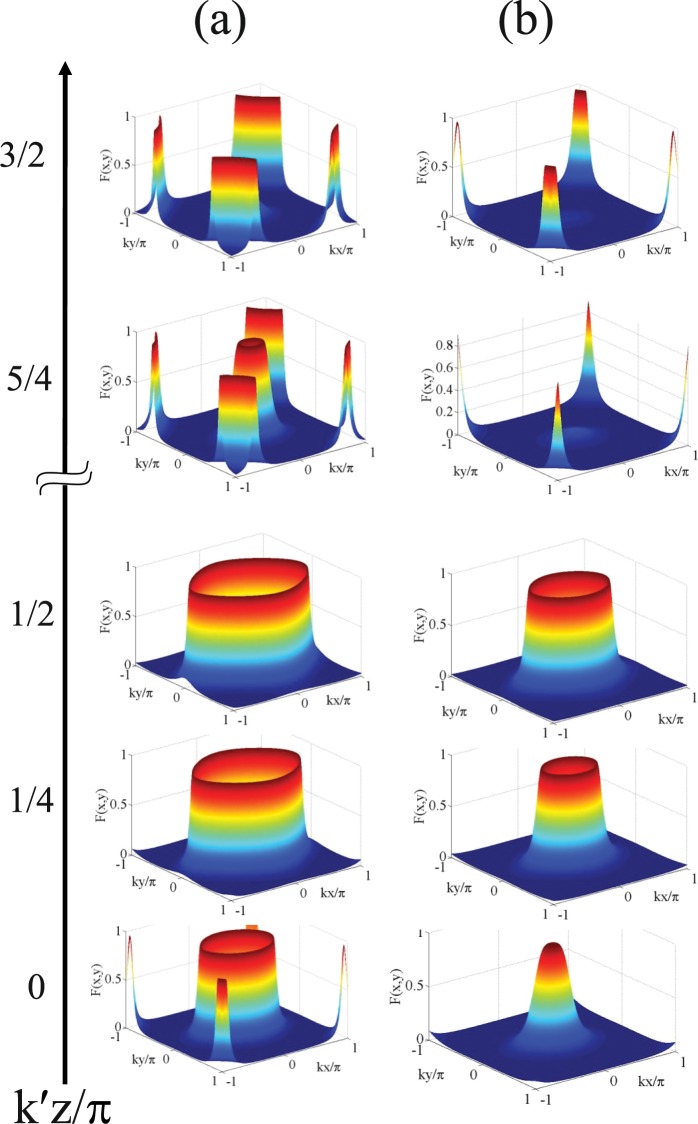
Plot of 2D Filter function $F(x,y)$ as a function of $(kx/\pi ,ky/\pi )$ for different $k^{\prime} z/\pi$ values: (**a**) ${R}_{1}={R}_{2}=7,{R}_{3}=5,{\eta }_{1}=0.9,{\eta }_{2}=0.7$ (**b**) ${R}_{1}={R}_{2}=5,{R}_{3}=3,{\eta }_{1}=0.9,{\eta }_{2}=0.7$. Other parameters for all the graphs: ${\gamma }_{23}=1,{\Lambda }_{1}=0.15={\Lambda }_{2}=0.15,{\omega }_{0}=10$, $\delta =0,\Delta =0,{\varphi }_{1}={\varphi }_{2}=\pi /2$ or −$\pi \mathrm{/2}$).

- Resonant case: Here, we have investigated the shape of the 2D Filter function $F(x,y)$ under the 2D resonant field arrangements (Eqs. 19 and 20) and the combined effect of fields, which act as a controlling knob to regulate localization probability and precision. Various spatial localization profiles are presented in Figs. 3, 4 and 5 with the parallel-axis and cross-axis configurations when the standing wave fields are at resonance ($\Delta =0$).

**Figure 6:**
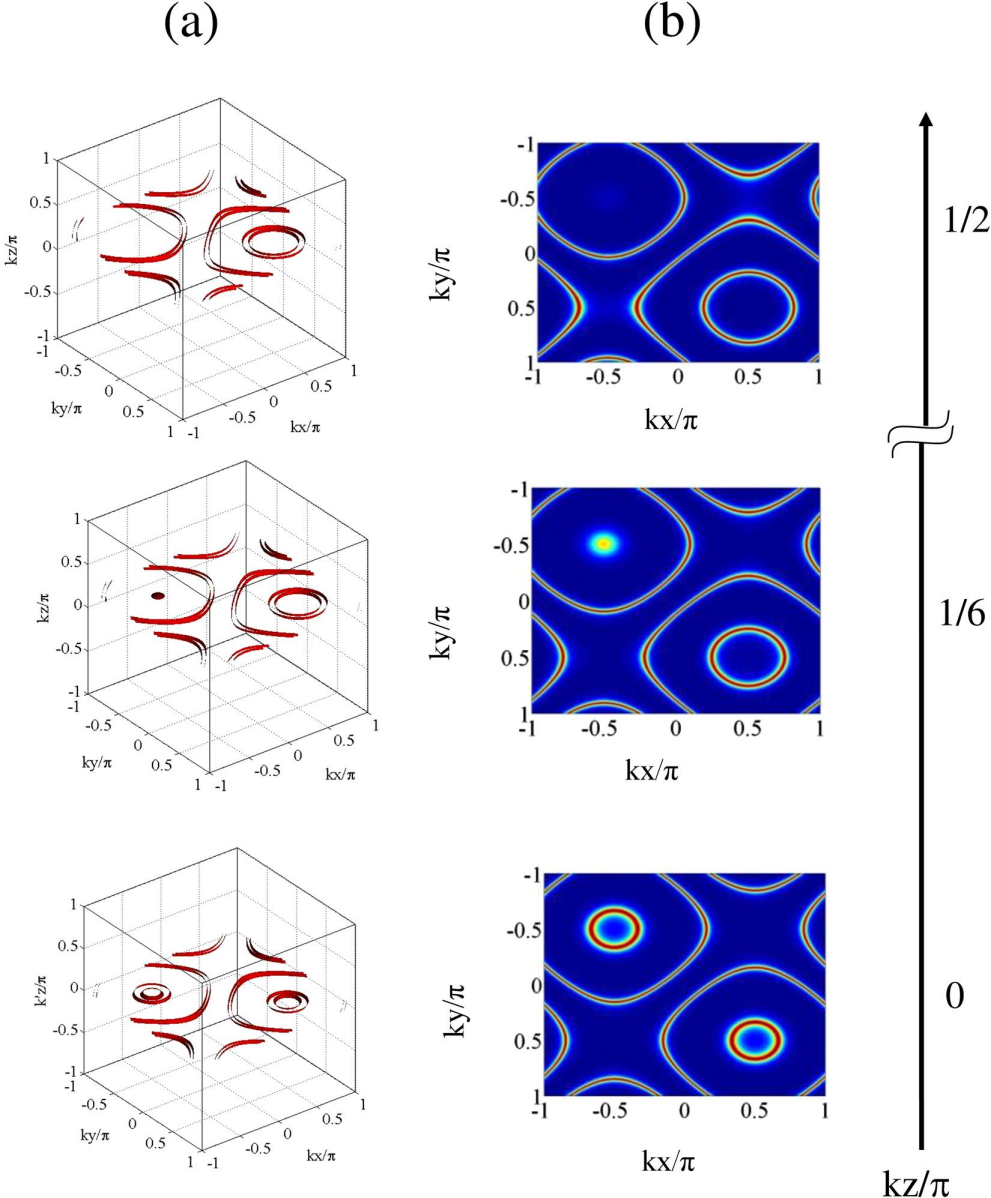
Comparison among the plots of (**a**) 3D Filter function $F(x,y,z)$ as a function of $(kx/\pi ,ky/\pi )$ for small ranges of $kz/\pi$ with respect of three specific values of $kz/\pi$ and (**b**) 2D Filter function $F(x,y)$ as a function of $(kx/\pi ,ky/\pi )$ for those $kz/\pi$ values: ${R}_{1}={R}_{2}=15,{R}_{3}=5,\delta =18,\Delta =0,{\gamma }_{23}=1$, ${\Lambda }_{1}=0.15,{\Lambda }_{2}=0.15,{\omega }_{0}=10,{\varphi }_{1}={\varphi }_{2}=0$.
- Parallel-axis Configuration: We explore 2D spatial localization profiles under the parallel-axis field arrangement with two orthogonal standing wave fields in resonant condition. We start from high Rabi frequencies and then sequentially decrease to lower values. But, for convenience of discussion of Fig. 3, we choose Fig. 3(c) to initiate an explanation. In the figure, the localization pattern appearing on one side looks like the mirror image of the other in the $kx/\pi -ky/\pi$ plane when the mirror is assumed to lie along the diagonal joining (−1, 1) and (1, −1). In each side, the localization pattern consisting of two discrete spikes and two concentric craters (with centers at the antinodes ((0.5, 0.5) and (−0.5, −0.5) of the standing-wave fields with ${R}_{1}={R}_{2}=7,\delta =3$) emerges as a result of the combined effect of spatially dependent four dynamic Stark-split components. Complete concentric crater-like structures originate from the symmetric contribution of these components, while the spike-like structures are consequences of their asymmetric contribution. With the increase of detuning parameter ($\delta =20$ and 15) and the Rabi frequencies ${R}_{1}$ and ${R}_{2}$ (${R}_{1}={R}_{2}=15$), asymmetric contribution dominates in the localization pattern and complete crater-like structures get fragmented as displayed in Fig. 3(a,b). We have verified that, the positions of craters in Fig. 3(c) are at the surface region surrounding the node-position in the plot of $|R(x,y)|$, while the position of spikes in that figure would correspond to the peripheral zone of anti-nodal surface relating to the side lobes of $|R(x,y)|$ plot. More specifically, when the atom is driven away from exact resonance in the standing-wave regime, the off-resonant atom-field interaction with an appreciable value of detuning leads to the asymmetric contribution of the dynamic Stark-split components and thereby dominates in the localization pattern.
  In Fig. 4 we present localization profiles where the localization probability and precision enhance with the selective parameter conditions. As presented in Fig. 4(a), for the values of Rabi frequencies ${R}_{1}={R}_{2}=7$, the criterion of atom-field off-resonance ($\delta =1$) condition in the orthogonal standing-wave regime results in two symmetric concentric craters without any spike in the first and third quadrants. At the condition of exact resonant interaction, for the same values of Rabi frequencies, as shown in Fig. 4(b), two single craters occur in the same quadrants due to the associative change in spatially modulated atom-field interaction. By reducing the values of Rabi frequencies (${R}_{1}={R}_{2}=5$), the separation between the analogous components resulted from dynamic Stark splitting gets reduced and, as a consequence, we obtain two Gaussian-like distribution structures (Fig. 4(c)).
- Cross-axis Configuration: With a view to attaining 100% detection probability of an atom in sub-wavelength 2D domain we resort to a different type of standing wave field arrangement (Eq. 20). With the insertion of the values of ${\phi }_{j}$ (${\varphi }_{1}={\varphi }_{2}=\pi /2$ or −$\pi \mathrm{/2}$) and ${\eta }_{j}$ (${\eta }_{1}={\eta }_{2}=0.7$), shifting occurs in the spatially modulated resonance and localization patterns evolve at the center of the surface of *xy*-plane with two concentric craters (Fig. 4(d)), single crater (Fig. 4(e)) and single Gaussian peak-like (Fig. 4(f)) structures. It has been checked that the positions of those localization patterns would correspond to the central zone of antinodal surface of $R(x,y)$-plots. The localization profile of Fig. 4(f) implies that an atom can be detected with 100% detection probability in sub-wavelength domain by using such a scheme and proper spatial phase condition.
  Another important display on the variation of localization patterns is presented in the presence of a third standing-wave field of Rabi frequency ${R}_{3}$, whose contribution is exploited here as an equivalent of a traveling-wave field. For this study, as mentioned earlier, a little change is employed in the field configuration under the cross-axis configuration (Eq. 20), which is expressed analytically as:
  23 $$R={R}_{1}\,{\sin }({\eta }_{1}kx+{\varphi }_{1})+{R}_{2}\,{\sin }({\eta }_{2}ky+{\varphi }_{2})+{R}_{3}\,{\sin }({\eta }_{3}{k}_{3}z)$$
  In this case, localization profiles are scanned by the detector placed along the *z*- direction at some discrete $k^{\prime} z$ $(k^{\prime} ={\eta }_{3}{k}_{3})$ values. This is aimed at obtaining the 2D-localization pattern with a variety of structures and with varying precision. Those localization patterns are presented in Fig. 5. For the combination of parameters as taken in Fig. 4(e) i.e., with the values of Rabi frequencies (${R}_{1}={R}_{2}=7,{R}_{3}=5$) in the resonant condition of spatial field and phases (${\varphi }_{1}={\varphi }_{2}=\pi /2$ or −$\pi \mathrm{/2}$) and $\delta =0$, the localization patterns are presented in Fig. 5(a) with the values of ${\eta }_{1}=0.9$ and ${\eta }_{2}=0.7$. All the patterns in Fig. 5(a) at $k^{\prime} z/\pi =0$ correspond to the null impact of ${R}_{3}$ field and are incorporated in the article to highlight the impact of component related to cross-configuration (${\eta }_{j}$) on 2D-localization structure. With the increase of ${\eta }_{1}$ from 0.85 to 1.0 keeping ${\eta }_{2}$ fixed at 0.7 or vice versa, the evolution of localization patterns, as is presented in Fig. 5(a), remains invariant. This range of tolerance of ${\eta }_{j}$ values indicates the robustness of the model. As is prominent from the graphs from $k^{\prime} z/\pi =0$ to 1, the projection of the localization surface on the $kx$/*π* - $ky$/*π* plane changes its shape from circular to elliptic with increasing eccentricities with the successive increment in the $k^{\prime} z/\pi$ values. Localization structures evolve through those with greater periphery signifying lesser probability of localization when they are scanned at growing values of $k^{\prime} z/\pi$ from 0 to 1. Same localization patterns repeat for $k^{\prime} z/\pi$ = 0 and 1 and also for the $k^{\prime} z/\pi$ values which are equidistant from 0 and 1 ($0 < k^{\prime} z/\pi  < 1$). The nature of localization profiles gets modulated for the $k^{\prime} z/\pi$ values beyond the value of unity. This is due to the negative influence of the third field (${R}_{3}$) in forming the localization patterns. This aspect is prominently visible in the 2D-localization figure shown for the values, $k^{\prime} z/\pi$ = 3/2, in comparison to that obtained for $k^{\prime} z/\pi$ = 5/4. More specifically, localization structure sets in with one crater-like pattern at the central node and fragmented spike-like pattern at all four edge nodes when the detector is placed at $k^{\prime} z/\pi$ = 5/4. When detector position is further increased to $k^{\prime} z/\pi$ = 3/2, the prominence of central localization structure almost vanishes. Here also, the localization patterns mimic each other when the detector is placed at the $k^{\prime} z/\pi$ values whose difference are same from 1 and 2. With the rise in $k^{\prime} z/\pi$ values, qualitative variations in localization profiles with distinctive features are evident in the graphs of Fig. 5(b). In Fig. 5(b) (${R}_{1}={R}_{2}=5,{R}_{3}=3$) we notice that the single spike-like structure with the same ${\eta }_{j}$ values assumed for Fig. 5(a), and other parameter conditions same as in Fig. 4(f), transforms into the elliptic crater-like localization shape when $k^{\prime} z/\pi$ varies zero to 1/2. For further higher values of $k^{\prime} z/\pi$, fragmented spike-like localization are seen at four edges with negligible localization probability at the centre. So far the localization precision is concerned, Fig. 5 contains its novelty in monitoring microscopic observation of 2D localization generated by spatially modulated coherence for quasi-continuous variation of the position of the detector along the *z*-axis.
- Non-resonant case: To obtain the essence of projection based 3D-localization leading to 2D-localization pattern in a plane, the different states of localization are scanned in the *xy*-plane by placing the detector at different locations along the $z$-axis. In this context, we have used the field arrangement in parallel-axis configuration with the resultant Rabi frequency expressed as:
  24 $$R={R}_{1}\,{\sin }(kx+{\varphi }_{1})+{R}_{2}\,{\sin }(ky+{\varphi }_{2})+{R}_{3}\,{\sin }(kz)$$
  where the fields are away from resonance (i.e. $\Delta \ne 0$). We have plotted the corresponding results in Fig. 6 and a comparison is presented between the plots of the 3D-presentation (Fig. 6(a)) of Filter function $F(x,y,z)$ for a small range of $kz/\pi$ values centred at a particular value of $kz/\pi$ with the corresponding 2D-projection (Fig. 6(b)) of the same in the *xy*-plane. For the particular value of $kz/\pi =0$, i.e., under the null contribution of the third field in spite of its presence, with parameters ${R}_{1}={R}_{2}=15;{R}_{3}=5;\delta =18$ in the non-resonant condition of spatial field, the localization feature as shown in Fig. 3(a) is slightly modified by the occurrence of inner crater-like patterns instead of spike-like patterns with centres in the first (0.5, 0.5) and third (−0.5, −0.5) quadrants (Fig. 6(a)). With the increase in $kz/\pi$ value to 1/6, a spike emerges at the third quadrant (−0.5, 0.5) with the replacement of the crater-like pattern whereas the periphery of the other crater-like localization feature in the first quadrant increases. For the increasing values of $kz/\pi$ from 1/4 to 1/3, the spike-like structure is getting disappeared gradually and finally it becomes inhibited at $kz/\pi =1/2$ resulting in large outer periphery of the crater-like structure in the first quadrant. It is interesting to point out that the proposed way of achieving the scope of position-dependent microscopy using the projection based technique shows novelty and robustness through the one-to-one correspondence of left and rights columns of Fig. 6.

**Figure 7:**
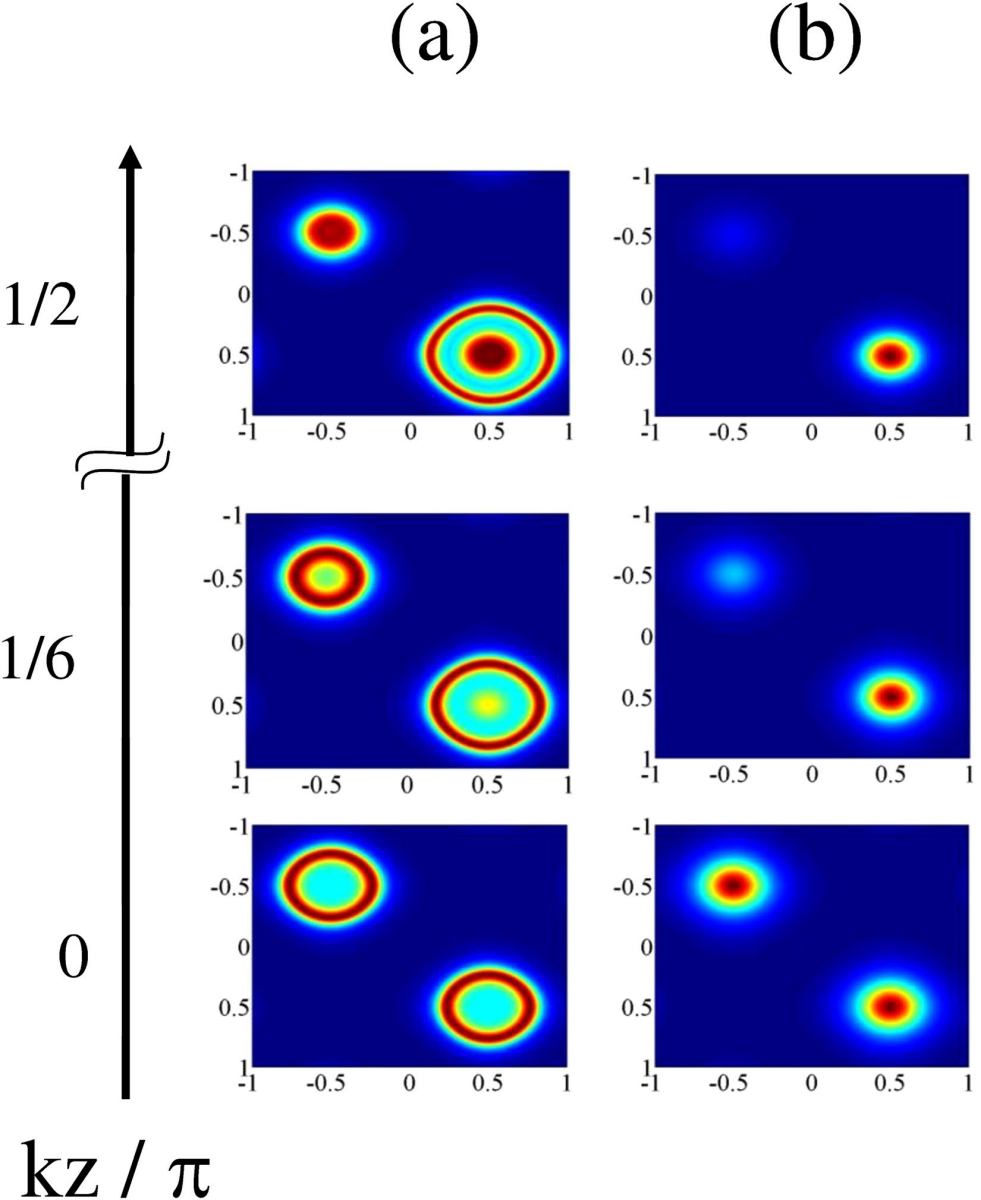
Variation in 2D Filter function $F(x,y)$ as a function of $(kx/\pi ,ky/\pi )$ for different $kz/\pi$ values: (**a**) ${R}_{1}={R}_{2}=5,{R}_{3}=1.5,\delta =0,\Delta =3$; (**b**) ${R}_{1}={R}_{2}=5,{R}_{3}=1,\delta =20,\Delta =3$. Other parameters ${\gamma }_{23}=1,{\Lambda }_{1}=0.15,{\Lambda }_{2}=0.15,{\omega }_{0}=10,{\varphi }_{1}={\varphi }_{2}=0$.
  For the extension of the present study in the direction of achieving the 100% detection probability of atom at the sub-half-wavelength regime, we have used the parameter conditions different from those given above and plotted the Filter function $F(x,y)$ projected on $({k}_{x}/\pi ,{k}_{y}/\pi )$ plane in Fig. 7 for the varying positions of the detector i.e., at different $kz/\pi$ values. In comparison to the variation of the $F(x,y)$ as obtained for the parametric conditions of Fig. 6, significant changes are made in the parametric conditions, ${R}_{1}={R}_{2}=5;{R}_{3}=1.5;\delta =0$, in the non-resonant case ($\Delta =3$) [Fig. 7(a)] when localization spectra are scanned at $kz/\pi$ = 0, 1/6, and then to 1/2. Two crater-like localization patterns emerge in the first and third quadrants without any contribution of ${R}_{3}$ field. As is seen for scanning at $kz/\pi =1/6$, one of them residing in the third quadrant gets transformed into a spike-like pattern with a small dip at (−0.5, −0.5) whereas another spike-like structure additionally appears at the coordinates (0.5, 0.5) within the crater having a bit larger circumference in the first quadrant. Finally, when scanned at $kz/\pi =1/2$, a spike-like pattern gets more pronounced having peak at (−0.5, −0.5) and the peak inside more larger periphery of the crater gains more localization probability at (0.5, 0.5). In Fig. 7(b) we attain the 100% detection probability for an atom in the sub-half-wavelength regime. In this respect, we set the detuning $\delta =20$ and two spike-like peak structures occur at centres of the first and third quadrants in the off-resonant condition of laser field, ${R}_{3}$. But, when its value is reduced to 1 with an increase in $kz/\pi =1/6$, the peak at (−0.5, −0.5) starts to die away and ultimately the spike-like structure only persists with peak at (0.5, 0.5) in the first quadrant with the complete suppression of the peak in the third quadrant at $kz/\pi =1/2$. Above results signify a strong correlation of localization structures with the scanning regime of the detector for which we intend to detect high-precision localization with 100% detection probability of atom at sub-half-wavelength domain.

**Figure 8:**
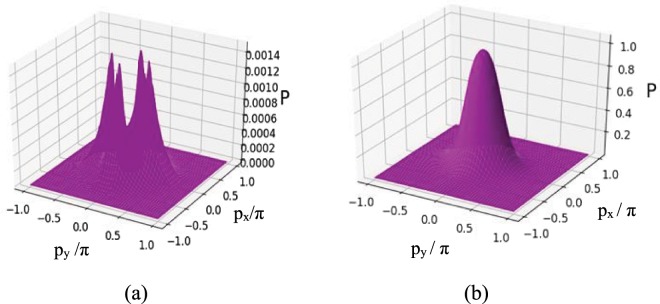
2D momentum distribution function P(p_x_,p_y_) plotted as a function of (p_x_/π,p_y_/π) in units of $\hslash /\lambda$: (**a**,**b**) correspond to the parameter conditions of Figs. 4(a) and 5(f).
  To comprehend the localization more clearly, we present the 2D momentum distribution probability ($P({p}_{x},{p}_{y})$) for the parameters of Figs. 3(a) in 8(a) and 4(f) in 8(b). The position-localization spectra corresponding to the parameters of Figs. 4(a) and 4(f) express the fact that the high precision and maximum probability of detection occurs for the second with respect to the first parameter condition. The momentum distribution graphs in Fig. 8(b) exhibit a central peak with maximum amplitude 570 times (approx.) of the four peaks of highly decreased amplitude obtained in Fig. 8(a). The graphs of Fig. 8(a) show that the peak height as well as peak width decrease in the momentum-distribution profile of the atom whose position localization is of very little precision.
  Another important point of investigation is to highlight that the numerical results corresponding to the graphs presented in Figs. 8(b) and 4(f) are in complete agreement with the uncertainty principle. As is consistent with the position-momentum uncertainty relation, a sharp localization peak in the position space must lead to a wider peak in the momentum space. The range of uncertainty for momentum in (${p}_{x},{p}_{y}$) space can be estimated by the following relations^25^:
  $$\frac{\Delta {p}_{x}}{{p}_{x}}\geqslant \frac{1}{2k\Delta x},\frac{\Delta {p}_{y}}{{p}_{y}}\geqslant \frac{1}{2k\Delta y},$$
  where the wave vectors along the $x$- as well as $y$- directions are represented by $k$. The above relations conform with the results derived from numerical simulations where the left-hand sides of the relations are 0.6 while the right-hand sides remain at the value of 0.5. It indicates that the greater is the precision for the position distribution of an atom, the lesser is the precision in the profile of its momentum distribution.

## Conclusion

In summary, we have adopted spatially-modulated-coherence controlled optical absorption microscopy to explore the technique of getting 2D-localization pattern in terms of projection-based 3D atom localization in a three-level atomic system at the microwave domain. Based on dynamic Stark effect in the absorptive response in a *V*-type atom we have resorted to different field configurations using spatially modulated standing wave fields. Several interesting localization features both in the 1D and 2D cases emerge, which indicate a strong correlation among localization structures and parameters associated with spatial field configurations. So far as the standing wave field is concerned, two different counter-propagating configurations (parallel and cross) serve effectively for the control of space localization of atom in the standing-wave regime. Our 1D results on localization envisage that the proposed scheme provides a promising way to obtain different types of localization structures with a varying number of peaks in sub-wavelength and sub-half-wavelength domains and finally attain 100% detection probability of the atom in the sub-half-wavelength regime through the proper tuning of traveling-wave field. Based on the cross-axis (parallel-axis) configuration adopted for studying 2D localization, the maximal localization probability in a one-wavelength range is made plausible with the generation of a single localization peak in the sub-wavelength (sub-half-wavelength) regime. It is interesting to highlight that the introduction of the third standing wave field in cross-axis configurations along the *z*-axis can provide the scope of scanning the atom with the desired localization probability and precision by placing the detector at a particular position along the *z*- axis. The evolution of the single-peak localization structure is found to be a direct consequence of introducing the third standing-wave field. Further, the freedom of varying the tuning parameters of cross-axis configuration in ensuring 2D-localization features with maximum detection probability makes the model substantial and novel. Without any cavity-induced field arrangement, our proposals with striking features in different localization schemes may give an impetus to the experimentalists to perform possible experimentation on high-precision atom localization through the fabrication of the devices for optical nanolithography.

## References

[1] Agarwal GS (2013). Quantum Optics.

[2] Storey P, Collett M, Walls DF (1992). Measurement-induced diffraction and interference of atoms. Phys. Rev. Lett..

[3] Thomas JE, Wang LJ (1995). Precision position measurement of moving atoms,’. Phys. Rep..

[4] Proite NA, Simmons ZJ, Yavuz DD (2011). Observation of atomic localization using electromagnetically induced transparency. Phys. Rev. A.

[5] Miles JA, Simmons ZJ, Yavuz DD (2011). Subwavelength localization of atomic excitation using electromagnetically induced transparency. Phys. Rev. A.

[6] Johnson KS (1998). Localization of metastable atom beams with optical standing waves: nanolithography at the Heisenberg limit. Science.

[7] Letokhov V (2007). Laser Control of Atoms and Molecules.

[8] Collins GP (1996). Gaseous Bose-Einstein Condensate Finally Observed. Phys. Today.

[9] Storey P, Collett M, Walls DF (1993). Atom-position resolution by quadrature-field measurement. Phys. Rev. A.

[10] Kunze S, Dieckmann K, Rempe G (1997). Diffraction of atoms from a measurement induced grating. Phys. Rev. Lett..

[11] Paspalakis E, Knight PL (2001). Localizing an atom via quantum interference. Phys. Rev. A.

[12] Ghafoor F, Qamar S, Zubairy MS (2002). Atom localization via phase and amplitude control of the driving field. Phys. Rev. A.

[13] Sahrai M, Tajalli H, Kapale KT, Zubairy MS (2005). Subwavelength atom localization via amplitude and phase control of the absorption spectrum. Phys. Rev. A.

[14] Agarwal GS, Kapale KT (2006). Subwavelength atom localization via coherent population trapping. J. Phys. B:At. Mol. Opt. Phys..

[15] Xu J, Hu X-M (2007). Localization of a two-level atom via the absorption spectrum. Phys. Lett. A.

[16] Ghafoor F (2011). Subwavelength atom localization via quantum coherence in a three-level atomic system. Phys. Rev. A.

[17] Dutta BK, Panchadhyayee P, Mahapatra PK (2012). Precise localization of a two-level atom by the superposition of two standing-wave fields. J. Opt. Soc. Am. B.

[18] Dutta BK, Panchadhyayee P, Mahapatra PK (2013). Coherent control of localization of a three-level atom by symmetric and asymmetric superpositions of two standing-wave fields. Laser Phys..

[19] Ding C, Li JH, Yang X, Zhan Z, Liu J-B (2011). Two-dimensional atom localization via a coherence-controlled absorption spectrum in an N-tripod-type five-level atomic system. J. Phys. B: At. Mol. Opt. Phys..

[20] Li JH, Yu R, Liu M, Ding C, Yang X (2011). Efficient two-dimensional atom localization via phasesensitive absorption spectrum in a radio-frequency-driven four-level atomic system. Phys. Lett. A.

[21] Zhang HT, Wang H, Wang Z (2011). Two-dimensional atom localization via two standing-wave fields in a four-level atomic system. Phys Scr..

[22] Rahmatullah, Qamar S (2013). Two-dimensional atom localization via probe-absorption spectrum. Phys. Rev. A.

[23] Wu JC, Ai BQ (2014). Two-dimensional sub-wavelength atom localization in an electromagnetically induced transparency atomic system. Eur. Phys. Lett..

[24] Jin LL, Sun H, Niu YP, Jin SQ, Gong SQ (2009). Two-dimension atom nano-lithograph via atom localization. J. Mod. Opt..

[25] Ding C, Li J-H, Yang X, Zhang D, Xiong H (2011). Proposal for efficient two-dimensional atom localization using probe absorption in a microwave-driven four-level atomic system. Phys. Rev. A.

[26] Shui T, Wang Z, Yu B (2015). Efficient two-dimensional atom localization via spontaneously generated coherence and incoherent pump. J. Opt. Soc. Am. B.

[27] Shui T (2018). High-precision two-dimensional atom localization from four-wave mixing in a double-Λ four-level atomic system. Laser Phys..

[28] Ivanov VS, Rozhdestvensky YV, Suominen KA (2014). Three-dimensional atom localization by laser fields in a four-level tripod system. Phys. Rev. A.

[29] Wang Z, Yu B (2015). Efficient three-dimensional atom localization via probe absorption. J. Opt. Soc. Am. B.

[30] Zhu Z, Chen A-X, Liu S, Yang W-X (2016). High-precision three-dimensional atom localization via three-wave mixing in V-type three-level atoms. Phys. Lett. A.

[31] Wang Z, Yu B (2016). High-precision three-dimensional atom localization via spontaneous emission in a four-level atomic system. Laser Phys. Lett..

[32] Ding C, Hao X, Jin R-B, Zhang D, Li J-H (2019). Optical localization of a single atom in three-dimensional space based on double-channel interaction. Laser Phys. Lett..

[33] Song F, Wang Z, Juan R, Yu B (2019). Atom localization in five-level atomic system driven by an additional incoherent pump. Appl. Phys. B.

[34] Thomas EF, Henriksen NE (2016). Non-resonant dynamic stark control of vibrational motion with optimized laser pulses. The Journal of Chemical Physics.

[35] Pierre, M., Sathyamoorthy, S. R., Svensson, I.-M., Johansson, G. & Delsing, P. Resonant and off-resonant microwave signal manipulation in coupled superconducting resonators. arXiv :1802.09034 (2018).

[36] Abdullah, N. R., Tang, C.-S., Manolescu, A. & Gudmundsson, V. The photocurrent generated by photon replica states of an off-resonantly coupled dot-cavity system. arXiv:1904.04888 (2019). 10.1038/s41598-019-51320-8 PMC6789108 31604993

[37] Ficek Z, Swain S (2005). Quantum Interference and Coherence: Theory and Experiments.

[38] Shui T, Yang W-X, Liu S, Li L, Zhu Z (2018). Asymmetric diffraction by atomic gratings with optical *PT* symmetry in the Raman-Nath regime. Phys. Rev. A.

[39] Shui T, Yang W-X, Li L, Wang X (2019). Lop-sided Raman–Nath diffraction in *PT*-antisymmetric atomic lattices. Opt. Lett..

[40] Panchadhyayee P, Dutta BK, Bayal I, Das N, Mahapatra PK (2019). Field-induced superposition effects on atom localization via resonance fluorescence spectrum. Phys. Scr..

